# Maxillofacial trauma and cerebrospinal fluid leak: a retrospective clinical study

**DOI:** 10.4314/ahs.v23i4.41

**Published:** 2023-12

**Authors:** Charles E Anyanechi, Birch D Saheeb

**Affiliations:** 1 Department of Oral and Maxillofacial Surgery, University of Calabar/University of Calabar Teaching Hospital Calabar, Nigeria; 2 Department of Oral and Maxillofacial Surgery, University of Benin/University of Benin Teaching Hospital Benin-City, Nigeria

**Keywords:** Craniofacial, trauma, fractures, cerebrospinal fluid, outcome

## Abstract

**Objectives:**

To determine the prevalence of maxillofacial fractures associated with persistent CSF leak, and to assess its bearing on clinical outcomes of consecutive patients managed at our centre.

**Methods:**

This was a retrospective cross-sectional study. The medical records of patients over 11-year period were analysed for age, gender, etiology of injuries, duration between injury and presentation to the hospital, types of facial fracture and their treatments, treatment done to control CSF leak, and complication(s). Descriptive and bivariate statistics were computed.

**Results:**

Overall, 1473 patients were evaluated, 66 (4.5%) presented with craniofacial injuries associated with persistent CSF leak after 5 days of non-surgical treatment. Males (92.5%, P= 0.0000) and those in the 21 to 30 years age group (59.1 %, P=0.01) were predominant. The most common (68.2%) type of fracture combination was Le Fort I, II and III, NOE, zygomatic complex and mandible. The commonest clinical presentation of CSF leak was rhinorrhea only, in 66.7% of patients (P= 0.001).

**Conclusions:**

This study shows that the prevalence of maxillofacial fractures associated with persistent CSF leak was low, which was 4.5% of patients that presented with persistent CSF leak and 84.9% of the cases resolved after treatment of the various maxillofacial fractures.

## Introduction

Cerebrospinal fluid (CSF) is a transparent fluid, produced by the ventriculocisternal portion of the nervous system, and serves as a shock absorber to protect the brain, cerebellum, and meninges. 1, 2 Any communication between the brain and facial structures leading to CSF leak is a pathological condition that can be life threatening. 3, 4 CSF leak or liquorrhoea commonly occurs following head trauma due to fronto-basal skull fractures. 1, 3 The condition associated with maxillofacial trauma presents clinically as rhinorrhea, orthorrhea or both. 2-4 The most causative factors differ from study to study, but have been shown to be due to direct trauma in 80 % of cases, intracranial surgery in 16 %, and of spontaneous etiology in 4 % of patients. 1, 3-5 Traumatic CSF leak may be caused by skull base fracture, and can also be a consequence of middle and upper facial fractures including naso-orbitoethmoidal fractures as a result of the compromise of the thin fovea ethmoidalis. 2, 6-8 The correlation between head injury including maxillofacial fractures, skull base fracture, and the resultant CSF leak is reported to be 2–30 %. 2, 3, 6, 9 CSF leaks occur if the bony cranial vault and its underlying dura are breached. Consequently, when maxillofacial fractures present clinically, the relationship of facial bones to skeletal anatomy of the skull base and cranium should be taken into consideration as complications like CSF leak can also occur due to the connection between the skull and nasal cavity or eustachian tube. 6, 9

CSF leakages directly occur from the anterior cranial fossa or indirectly from the middle or posterior cranial fossa through eustachian tube. 1-3 The most frequent scenario being direct anterior cranial fossa leak due to the firm attachment of the lamina dura to the thin cribriform plate and ethmoid bone. 1, 2

A detailed computed tomography (CT) scan is mandatory in all patients if CSF leak is suspected. Chemical tests confirming the CSF leak include beta transferrin and intrathecal fluorescein. 10, 11 Apart from clinical diagnosis, β2-transferrin is highly sensitive and specific for recognizing CSF, and is the laboratory test of choice. 12 The treatment of CSF leak is by either conservative therapies or surgical interventions. 13, 14 This condition among other complications it can elicit, may lead to meningitis, recurrent CSF leak after treatment or even death if not properly managed. 1, 2, 3, 9

Post-traumatic CSF leak after craniofacial injuries remains a challenge for cranio-maxillofacial surgeons due to the fatal complications that might follow if not properly managed. 15 Majority of studies done earlier in this category of patients have at times provided discordant results in terms of the prevalence, etiology, pattern of presentation and treatment outcome. The purpose of this study is to determine the prevalence of maxillofacial fractures associated with persistent CSF leak, and to assess its bearing on the clinical outcomes of consecutive patients presenting to a tertiary health facility.

## Materials and methods

The medical records of patients in our center over an 11-year period were evaluated for demographic and clinical characteristics related to the etiology and management of craniofacial injuries associated with persistent CSF leak following traumatic injury. This was a retrospective cross-sectional study of patients who sustained simultaneous maxillofacial and cranial injuries associated with persistent CSF leak in form of rhinorrhea and/or otorrhea, in order to determine their prevalence and clinical characteristics. The injuries include facial fractures extending into the intracranial space injuring dura. Persistent CSF leak is defined by drainage more than 5 days after injury. 13 The patients presented at the Accident and Emergency Department and Oral and Maxillofacial Surgery Clinic of our institution. The study was exempted from Ethical clearance by the institution's Regional Ethics Review Board because of its retrospective design, but informed consent was obtained from each patient before any surgical procedure was undertaken. Following the injury and presentation to the study center, and suspicion of CSF flow, the patients were initially observed clinically for 5 days to see if the CSF leak will stop spontaneously. All the patients were covered with broad-spectrum antibiotics. During this period, non-surgical therapy was instituted immediately with patients' head elevated in recumbent position, and strict sinus precautions to minimize increases in intracranial pressure, including straining, sneezing and blowing of the nose, and bed rest were observed. However, where this expectation failed, treatment of the underlying cause was then carried out to arrest this pathological condition. What determined the waiting period before initiation of definitive treatments for each patient was partly clinical deterioration in terms of neurological deficits among other confounding variables.

The diagnosis of the craniofacial injuries and CSF leak were based on the clinical data obtained from the medical records of the patients, including relevant radiological images particularly computed tomography (CT) scan of head including the jaws and test of the fluid for the presence of β2-transferrin if available. In addition, those cases that presented with otorrhea, pneumatic otoscopy was performed to exclude the presence of middle ear fluid.

Cases included in the study are:

- Patients who sustained simultaneous maxillofacial and cranial injuries associated with persistent CSF leak in form of rhinorrhea and/or otorrhea which lasted beyond 5 days and was arrested by definitive treatment of underlying cause.
- Patients that are non-smokers of tobacco and narcotic drugs.
- Those not on steroid therapy.
- Patients having no medical conditions that compromise treatment and healing process.
- Subjects with complete medical records.
- Those who kept a minimum of 3.5 years follow-up appointments.

Those excluded were patients who sustained simultaneous maxillofacial and cranial injuries associated with persistent CSF leak in form of rhinorrhea and/or otorrhea but:

- With compromised medical history.
- Failed to attend postoperative follow-up visits for a minimum period of 3.5 years.
- With CSF leak that stopped spontaneously within the 5 days period of observation.
- Died before commencement of definitive treatment.
- With incomplete data.
- Definitive treatment started less than 5 days after injury.

The surgical procedures for treatment of the maxillofacial injuries were done under standard surgical protocols except that closed reduction and non-rigid fixation were used to treat fractures. The primary predictor of CSF leak was the presence of cranial and maxillofacial injuries while the outcome variables were occurrence of complications related to the treatment of cranio-facial injuries. Clinical records of the patients over the 11-year period from January 2010 through December 2020 were analysed for age, gender, etiology of injuries, duration between injury and presentation to the hospital, types of facial fractures and their treatments, treatment done to control CSF leak, and complication(s) related to the treatment of craniofacial injuries. Subsequently, retrospective chart review was used for the compilation of data of interest.

Complication was diagnosed based on patients' complaints and clinical and radiological evaluation during follow-up period. The outcome of treatment was determined by the presence or absence of pain, particularly when the jaws are functioning, meningitis, late CSF leak in form of rhinorrhea, otorrhea or both, and death during management of the patients including non-union and mal-union of maxillofacial fractures, and mal-occlusion. Meningitis was diagnosed if patient presented clinically with high fever and neck stiffness in the course of management. The presence or absence of non-union, mal-union and mal-occlusion were determined for each patient on the 6th and 8th week after commencement of treatment of maxillofacial fractures.

The data obtained were analysed with EPI Info 7, 2012 software (US Centers for Disease Control and Prevention, Atlanta, GA, USA). For analyses, descriptive statistics and tests of significance, Chi-square and Fisher's Exact were computed. P-values <0.05 were considered significant.

## Result

Overall, 1473 patients who had craniofacial injuries were evaluated during the study period. However, 66 (4.5%) patients presented with persistent CSF leak beyond 5 days after sustaining the injury. The cranial injuries sustained by the patients were classified as craniocerebral, and were managed by the neurosurgeons.

The distribution of age and gender of the 66 subjects are shown in table 1.

**Table 1:** Age and gender distribution of the 66 patients

	Gender					
Age group	Male		Female		Total	
	n	%	n	%	n	%
11-20	2	3.0	0	0	2	3.0
21-30	36	54.6	3	4.5	39	59.1
31-40	11	16.7	1	1.5	12	18.2
41-50	7	10.6	0	0	7	10.6
51-60	4	6.1	1	1.5	5	7.6
61-70	1	1.5	0	0	1	1.5
**Total**	61	92.5	5	7.5	66	100.0

Age: χ^2^= 118, df=10, P=0.01

Gender: χ^2^= 118, df=10, P=0.0000

Their ages ranged from 18 to 64 years (mean 37.8 ± 11.3 years) while patients in the age category of 21 to 30 years (59.1 %, P= 0.01) were more than the other groups. The male to female ratio was 12.2:1, and the males (P= 0.0000) outnumbered the females in all age categories.

All the injuries were caused by road traffic accidents (RTA), motor vehicle (n= 46, 69.7%) and motorcycle related, 20 (30.3%). After the injuries, the subjects presented to the hospital between 3.2 to 72.4h (mean 38.3 ± 0.4h). The different types of fractures and their combinations, in addition to their distribution according to the age categories of patients are shown in table 2.

**Table 2:** Distribution of patients' age according to types of facial fractures

Types of fractures	Age of patients						
	11-20	21-30	31-40	41-50	51-60	61-70	Total (%)
Le Fort I, II, III, NOE, zygomatic complex, mandible	0	16	4	3	1	0	24(36.4)
Le Fort II, III, zygomatic complex, NOE, mandible	0	12	5	2	2	0	21(31.8)
Le Fort I, II, NOE, frontal sinus	2	9	3	1	0	1	16(24.2)
Naso-orbito-ethmoidal, frontal sinus	0	2	0	1	2	0	5(7.6)
**Total**	2	39	12	7	5	1	66(100.0)

χ^2^= 110, df= 8, P= 0.001

### NB: NOE= Naso-orbito-ethmoidal

Fracture combinations of Le Fort I, II, III/NOE/zygomatic complex/ mandible and Le Fort II, III/NOE/zygomatic complex/mandible including Le Fort I, II/naso-orbito-ethmoidal/frontal sinus were more frequent than Naso-orbito-ethmoidal/frontal sinus (P= 0.001, Tables 2 and 3). Also, the distribution of facial fractures in relation to the symptoms of CSF leak revealed rhinorrhea only as significant relative to other clinical presentations (P= 0.001, Table 3).

**Table 3:** Distribution of facial fractures sustained by the patients in relation to symptoms of CSF leak

Types of fractures	Rhinorrhea	Otorrhea	Rhinorrhea/Otorrhea	No. of patients
	n(%)	n(%)	n(%)	Total (%)
Le Fort I, II, III, NOE, zygomatic complex, mandible	14(21.2)	5(7.6%)	5(7.6)	24(36.4)
Le Fort II, III, zygomatic complex, NOE, mandible	16(24.2)	3(4.6)	2(3.0)	21(31.8)
Le Fort I, II, NOE, frontal sinus	10(15.1)	0(0)	6(9.1)	16(24.2)
Naso-orbito-ethmoidal, frontal sinus	4(6.1)	1(1.5)	0(0)	5(7.6)
**Total**	44(66.6%)	9(13.7)	13(19.7)	66(100.0)

χ^2^= 118, df= 10, P= 0.001

The categorization of age of patients according to symptoms of CSF leak shows the 21-30 years group was more in frequency relative to other age groups (P= 0.01, Table 4).

**Table 4:** Distribution of age of patients according to symptoms of CSF leak

Age groups	Rhinorrhea	Otorrhea	Rhinorrhea/Otorrhea	Total	(%)
11-20	2	0	0	2	3.0
21-30	32	1	6	39	59.1
31-40	6	4	2	12	18.2
41-50	1	3	3	7	10.6
51-60	2	1	2	5	7.6
61-70	1	0	0	1	1.5
**Total**	44(66.7)	9(13.6)	13(19.7)	66	100.0

χ^2^= 110, df= 8, P= 0.01

All subjects in this study experienced successful resolution of CSF rhinorrhea and/or otorrhea using a variable combination of closed reduction with MMF, CSF diversion via lumbar subarachnoid drainage, and dural repair with mucosal graft (Table 5). However, majority of the cases (n= 56, 84.9%, P= 0.000, Table 5) resolved after treatment of the facial fractures.

**Table 5:** Distribution of treatment done to arrest CSF leak in the 66 patients

Types of fractures				
	*CR	†LD	‡DR	Total (%)
Le Fort I, II, III, NOE, zygomatic complex, mandible	19(28.8)	4(6.1)	1(1.5)	24(36.4)
Le Fort II, III, zygomatic complex, NOE, mandible	20(30.3)	1(1.5)	0(0)	21(31.8)
Le Fort I, II, NOE, frontal sinus	15(22.8)	1(1.5)	0(0)	16(24.2)
Naso-orbito-ethmoidal, frontal sinus	2(3.0)	2(3.0)	1(1.5)	5(7.6)
**Total**	56(84.9)	8(12.1)	2(3.0)	66(100.0)

χ^2^= 118, df= 10, P= 0.000

* Closed reduction with MMF

† CSF diversion via lumbar subarachnoid drainage

‡ Dural repair with mucosal graft

The CSF leak was arrested in the patients treated for facial fractures within 24 to 72h (mean 37.3 ± 1.4h) after the procedure. The facial fractures were managed by closed reduction techniques including maxillo-mandibular fixation (MMF) where appropriate. The period of MMF ranged from 4 to 6 weeks. In the other patients, persistent CSF leaks were managed by CSF diversion via lumbar subarachnoid drainage for 7 to 10 days, while intracranial procedures by dural repair with mucosal graft were done after lumbar drainage failed to resolve the leak after 10 days. Following the dural repair with mucosal graft in the two patients, the CSF leak stopped within 24h. Follow-up of patients ranged from 3.5 to 9.3 years (mean 6.7 ± 3.2 years). During this period, there was no evidence of pain, malocclusion, non-union and mal-union of maxillofacial fractures, in addition to meningitis, recurrent CSF leak or death among the patients.

## Discussion

This study showed 4.5% of patients presented with persistent CSF leak related to craniofacial injuries, and majority (84.9%) of the cases resolved after treatment of the facial fractures. In studies of this nature, it is difficult to compare results obtained with earlier investigators because of the differences in study designs. However, the prevalence obtained in this study is within the range documented by Conforti et al., 6 and Pappachan and Alexander. 9 On the contrary, it is higher than the 1.3% obtained by Hasheminia et al., 4 but lower than that of Namet al., 16 who reported traumatic craniocerebral injuries occurring in 12-45.5% of maxillofacial fractures. The low prevalence obtained may be related to the ban placed on the use of motor-cycle for public transportation in the study community more than a decade ago. 17

The age and gender distribution of patients in this study is consistent with most reports in the available literature. 4, 6, 9 This could be due to males particularly the younger age group who are more involved in outdoor activities, thus they are more prone to accidents and traumatic events. However, Bell et al., 13 showed higher prevalence of facial fractures in males but a higher percentage of CSF leak amongst injured females.

All the cases reported in this study were caused by RTA, due to motor vehicle and motorcycle related accidents. Most other previous studies recorded RTA as the major cause, in addition to other etiological factors like falls from reasonable heights. 4, 18-20 Similar to the present study, Pappachan and Alexander 9 noted that accidents from motorcyclists without helmets contributed significantly to the etiology of cases in their series.

Cranial and facial fractures following head injury are very common. As documented earlier and reported in the present study, vulnerable sites of head injuries include the cranium, orbit, zygoma, nose, maxilla, and mandible.^1^, ^3^, ^6^, ^16^ CSF leak was observed most frequently in patients with fracture in the maxilla and zygoma, and all cases had skull base fracture as well. This is similar to the report of some other earlier researchers. 4, 13 The facial fractures recorded in this study have been reported earlier and show that fractures involving the middle third of the face are more susceptible to it. 1, 4, 16 As noted by some authors, 3, 6, 9, 16 CSF leak is more common with anterior skull base fractures associated with Le Fort II, III and in frontal bone fractures which is due to the firm attachment of the dura to the anterior skull base. Furthermore, depressed maxillofacial injuries fracture the cribriform plate of ethmoid bone, which fractures more easily than frontal, sphenoid and temporal bones because of its thin and fragile nature. 3, 16 It has been shown that as the fracture occurs at sites closer to the central nervous system (CNS), the chance of CSF leak gets higher. 4, 5, 16 The disruption usually occurs lateral to the cribriform plate but can also be due to disruption of the sphenoidal, ethmoidal, and frontal sinuses producing a dural tear and communication with the subarachnoid space. As shown in this study, CSF leak can be expressed as rhinorrhea or otorrhea, which is due to the connection between the skull and nasal cavity or eustachian tube. 3, 6, 16 Consequently, cranio-maxillofacial injuries can lead to a communication between the intracranial cavity and surrounding facial structures, increasing the intracranial pressure (ICP) that manifests as CSF rhinorrhea, otorrhea and orbitorrhea. 16, 18 Similarly, fractures involving the frontal or basilar skull may lacerate the dura, allowing CSF to exit from the nose, or fractures with dural laceration may allow air to enter the intracranial area causing pneumocephalus. 1, 16, 18 Either condition permits entry of organisms through the meninges with the possibility of meningitis. Consequently, cranio-facial traumas may be associated with contamination of wounds with various virulent pathogens such as streptococcus pneumonia and haemophilus influenzae. 6, 16 The traumatic formation of this connection can be life threatening and could make a communication between the nasal cavity and paranasal sinuses with cranium, which can cause meningitis, encephalitis or an ascending infection inside the skull. 3, 6, 18 This was not encountered in the present study.

This study shows that the most common form of clinical presentation was rhinorrhea which is similar to most other reports. 1, 4, 10 However, the frequency of patients with CSF otorrhea compared to rhinorrhea was reported to be higher in the study by Bell et al. 13 These variations in presentation may be due to the predominant type of cranio-facial injuries sustained in each study which may be related to the etiological factors causing the injuries. In this study, CSF leakage was observed immediately after trauma, but this can sometimes be delayed and observed days to weeks after the traumatic event. Some researchers report symptoms of CSF leak as early as 2 days or can be seen after 3 months of injury. 3, 6 This situation may occur when debris such as a blood clot or avulsed tissue obstructs free passage of the CSF, leading to late leakage which occur due to clot lysis or an increase in intracranial pressure. On the contrary, CSF leak was observed immediately the patients presented in this study.

Most times, CSF is mixed with blood, lacrimal and nasal secretions making its detection difficult. Clear CSF is collected in a vial, and an absence of sediment and a glucose level of approximately 45 mg/dL is usually suspicious.^10^, ^21^ It has been suggested that, the double-ring sign, absorption of blood and CSF onto a paper towel, produces a small, central blood ring with a large, peripheral, clearer fluid ring surrounding it. 10, 20, 21 Furthermore, in the presence of a skull base fracture on computed tomography and a clinical CSF leak, there is usually no need for a further confirmatory test. 20, 21 In cases where a confirmatory test is needed, the beta-2 transferrin assay is the test of choice because of its high sensitivity and specificity which will be positive for CSF but negative for other confounding fluid like thin mucus. 10, 21 In the present study, β2-transferrin assay was used to confirm the presence of CSF leak where there was doubt. Quantitative comparison of glucose levels of the fluid with that of serum and nasal secretions is also a method that can be used but carries a high false-positive rate and does not consistently differ between CSF and secretions of allergic rhinitis. 21 Consequently, β2-transferrin is sensitive and specific for diagnosing CSF, and is the preferred chemical test. 10, 12

In the present study, it can be argued that those patients (84.9%) that recovered after treatment of facial fractures did so spontaneously or because the procedures facilitated the closure of the Dural tear and communication, whereas 12.1% and 3.0% needed lumbar diversion via lumbar subarachnoid drainage and intracranial surgical procedure (dural repair with mucosal graft) respectively for the arrest of the CSF leak. It is possible that reducing fracture fragments can indirectly restore Dural integrity and stop CSF leakage. 9, 16 The presence of a Dural tear is not a contraindication to surgical repair of mandibular and midfacial fractures. 3, 6, 16 Early reduction and fixation will likely reduce the changes in intracranial pressure associated with mobile fractures that often results in intermittent pumping of CSF through a Dural disruption. 3, 6 Some authors suggested that if CSF leakages have not subsided in 3 to 4 weeks postreduction, surgical correction of the leak is usually indicated. 3, 6, 9

The literature suggests that for those cases that recover spontaneously, careful evaluations of defects is important, because a transient leak may have stopped as a result of herniated brain, and late complications, such as meningitis or death, may occur if these are left untreated. 22-26 The risk of meningitis secondary to a conservatively managed Dural tear is significant and adequate follow-up is important. 3, 6 Some researchers also suggest early exploration when CSF rhinorrhea is encountered in the presence of trauma. 25, 26 The outcome of this study is closely similar to the reports of some earlier researchers. 2, 13, 16, 19 However, in the study by Hasheminia et al., 4 47% cases recovered spontaneously, 11.8 % were treated after lumbar drainage, and 41.2% were surgically managed. The variations in the treatments used to arrest the CSF leak might be due to the type, severity of the injuries or the size of the skull base defects which suggest that as the injuries or defects become more severe, the protocol tends toward surgical intervention. In contemporary practice, where the facilities are available and if the defect is not too large, endoscopic endonasal management has also proved useful and reliable. 22, 23 The decision to carry out intracranial approach is due to the large size of skull base defects and unavailability of endoscopic instruments in the study center. However, intracranial approach remains a reliable procedure for Dural repair. Treatment decisions should be dictated by the severity of neurological decline and the presence or absence of associated intracranial lesions. 24 The timing for CSF drainage and surgical procedures must be decided with great care and with a clear strategy. 24, 25

Following injury and subsequent treatments, no complication was recorded during follow-up. Though, the period of follow-up in this study may be regarded as short as complication has been reported decades after the initial traumatic craniofacial injury. 26 In addition, the presence of craniocerebral injuries sometimes leads to delays in treatment of maxillofacial fractures and may actually increase the risk of complication. 25, 26 Clinical evidence shows that the longer a CSF leak persists, the greater the risk of meningitis. 24-26 However, early intervention in the treatment of maxillofacial fractures in the present study may have contributed in preventing complications as most of the patients presented early in the hospital.

Post-traumatic CSF leak after craniofacial injuries remains a challenge for cranio-maxillofacial surgeons due to the high morbidity and fatal complications that might follow if not properly managed. In the present study, patients presented early for clinical evaluation and treatment, presenting less management challenges. This study has determined the prevalence of maxillofacial fractures associated with persistent CSF leak, in addition to their bearing on the clinical outcomes of consecutive patients in this community. This will enhance the recognition of the significance of universal coverage and equity in health services by the agencies concerned in the study environment in order to improve the facilities and manpower on ground for the management of these cases.

It is possible that not all the patients afflicted with this condition in this community presented for clinical evaluation and treatment during the study period. This would have negatively affected the prevalence rate obtained. Closed reduction technique was used to manage the maxillofacial fractures; however, the current concept is the use of rigid internal fixation which improves patients' wellbeing post-operatively, and minimizes discomfort and complications, particularly occlusal discrepancies after treatment. Closed reductions with MMF are less tolerable to patients and reduce the vital capacity of the lungs, in addition to aggravating respiratory insufficiency. 27, 28 Also, the treatment of these patients was done by different surgeons with differing surgical competences. This could as well have introduced some discrepancies that might have affected the treatment outcome. It is also possible that some patients who had complications failed to report back to the hospital for management. These patients may have opted for alternative means of treatment or decided to live with their conditions.

## Conclusions

This study shows that the prevalence of maxillofacial fractures associated with persistent CSF leak was low, which was 4.5% of patients that presented with persistent CSF leak and 84.9% of the cases resolved after treatment of the various maxillofacial fractures. This suggests that prompt management of these patients when they present early for treatment enhances good treatment outcome, and should be encouraged. Post-traumatic CSF leak after craniofacial fractures still remains a challenge for cranio-maxillofacial surgeons due to the possible fatal complications that may accompany it.
